# Safety and Efficacy of High-Dose Vitamin B6 as an Adjunctive Treatment for Antipsychotic-Induced Hyperprolactinemia in Male Patients With Treatment-Resistant Schizophrenia

**DOI:** 10.3389/fpsyt.2021.681418

**Published:** 2021-08-26

**Authors:** Chuanjun Zhuo, Yong Xu, Haibo Wang, Tao Fang, Jiayue Chen, Chunhua Zhou, Qianchen Li, Jie Liu, Shuli Xu, Cong Yao, Weiliang Yang, Anqu Yang, Bo Li, Yuhui Chen, Hongjun Tian, Chongguang Lin

**Affiliations:** ^1^Key Laboratory of Multiple Organ Damages of Major Psychoses (MODMP_Lab), Tianjin Fourth Center Hospital, The Fourth Central Hospital Affiliated With Nankai University, The Fourth Central Hospital Affiliated to Tianjin Medical University, Tianjin, China; ^2^Key Laboratory of Real Time Brain Circuit Tracing in Neurology and Psychiatry (RTBNP_Lab), Tianjin Fourth Center Hospital, The Fourth Central Hospital Affiliated With Nankai University, The Fourth Central Hospital Affiliated to Tianjin Medical University, Tianjin, China; ^3^Department of Psychiatry, First Hospital/First Clinical Medical College of Shanxi Medical University, Taiyuan, China; ^4^Mental Disorder Therapy Center for Cognitive Impairment and Sleep Disorders, First Hospital of Shanxi Medical University, Taiyuan, China; ^5^Peking University Clinical Research Institute, Peking University First Hospital, Beijing, China; ^6^Laboratory of Neuro-Imaging and Comorbidity (PNGC_Lab), Tianjin Anding Hospital Affiliated to Nankai University, Tianjin Medical University, Tianjin, China; ^7^Department of Pharmacology, The First Hospital Affiliated to Hebei Medical University, Shijiazhuang, China; ^8^Department of Treatment Resistant Schizophrenia, Tianjin Kangtai Hospital, Tianjin, China; ^9^Department of Psychiatry, Wenzhou Seventh Peoples Hospital, Wenzhou, China

**Keywords:** TRS, aripiprazole, drug repurposing, cognitive ability, PANSS

## Abstract

This study aimed to investigate the safety and efficacy of high-dose vitamin B6 (vB6) as an adjunct treatment for antipsychotic-induced hyperprolactinemia (AIHP) in male patients with treatment-resistant schizophrenia (TRS). In this randomized double-blinded controlled study, patients were randomized (1:1) into a control group given aripiprazole (ARI; 10 mg/day; *n* = 100) or an intervention group given vB6 (300 mg/12 h for 16 weeks; *n* = 100). Prolactin levels, psychotic symptoms [Positive and Negative Syndrome Scale (PANSS)], cognitive function [MATRICS Consensus Cognitive Battery (MCCB)], liver function, kidney function, growth hormone level, micronutrient levels, blood lipids, and adverse secondary effects (ASEs)[Treatment Emergent Symptom Scale (TESS) and Barnes-Akathisia scale] were monitored. After a 16-week treatment period, the vB6 group showed a 68.1% reduction in serum prolactin levels (from 95.52 ± 6.30 μg/L to 30.43 ± 18.65 μg/L) while the ARI group showed only a 37.4% reduction (from 89.07 ± 3.59 μg/L to 55.78 ± 7.39 μg/L). During weeks 1–4, both treatments reduced prolactin similarly. Subsequently, the ARI effect plateaued, while the vB6 effect remained robust. The vB6 group showed better alleviation of psychotic symptoms and cognitive impairment. No serious ASEs were observed; ASEs were more frequent in the ARI group. AIHP reduction efficacy of vB6 was associated with baseline prolactin and triglyceride levels, total vB6 dosage, and education level. In conclusion, compared with the ARI group, TRS patients given vB6 showed better attenuation of AIHP, lower ASE scores, and greater improvements in clinical symptoms and cognitive impairments. These results support further consideration of vB6 as a putative treatment for AIHP.

**Trial Registration:** ChiCTR1800014755

## Introduction

Antipsychotic-induced hyperprolactinemia (AIHP) is a difficult to manage secondary adverse effect (ASE) of antipsychotic medication use (1, 2). The etiology of AIHP is unclear (3–8). The main proposed mechanisms of the pathogenesis of AIHP are the estrogen protection hypothesis and the stress prolactin-dopamine hypothesis (5–7), both of which involve alterations in dopamine receptor activation (5–7). Each of these hypotheses provides only a partial explanation. Accordingly, the need for more studies examining functional disturbances of the hypothalamic-pituitary-gonadal axis in patients with psychosis has been emphasized (5–7). The clinical manifestations of hyperprolactinemia, including sexual dysfunction and gynecomastia in male patients, can cause patients to feel distressed and to suffer a reduction of life quality (4–6, 8) and thus result in reduced compliance with antipsychotic treatment adherence and, ultimately, a deteriorated prognosis (9, 10).

Several decades of research have not produced complete alleviation of AIHP (2, 11–13), especially in patients with treatment-resistant schizophrenia (TRS). In most cases, patients with TRS need antipsychotic dosages two to three times the regular dosage regime and may still suffer from clinically significant residual symptoms (14). The long-term and high-dosage characteristics of their antipsychotic treatment increases the risk of serious ASEs, including difficult to treat hyperprolactinemia (15, 16). In an effort to alleviate AIHP, many physicians will switch patients from high-dosage antipsychotics to low-dosage aripiprazole (ARI) (17, 18), which has been suggested to alleviate the condition in at least some cases (19, 20), albeit not without some controversy (20–24). Recommendations for reducing the risk of or alleviating AIHP, beyond low-dosage ARI, including metformin, traditional Chinese herbs, and low-dosage dopamine agonists (e.g., bromocriptine) (17, 21). However, these approaches are not highly satisfactory, especially with respect to the management of psychotic symptoms.

Some older studies examining the effects of the water-soluble micronutrient vitamin B6 (vB6), may be relevant to the problem of AIHP. In 1977, Delitala et al. found 600-mg vB6 injections reduced prolactin levels in patients using antipsychotics and reported a pharmacodynamic curve of the resultant altered prolactin levels over several hours (21). In a 1978 study of lactating women, de Waal et al. found that vB6 (300–1,200 mg) did not reduce chlorpromazine-induced hyperprolactinemia, but perhaps this negative conclusion should be re-considered given that the women were actively producing milk, a physiological process that requires elevated prolactin (22). Indeed, in 1979, Rosenberg et al. reported that vB6 reduced chlorpromazine-induced hyperprolactinemia in animal models (25). Following these studies, however, the safety and potential efficacy of vB6 for reducing elevated prolactin levels in patients taking antipsychotics were not established.

Patients have been given vB6 to alleviate nausea and vomiting (due to pregnancy or radiation sickness) (25) and to alleviate premenstrual/menstrual breast pain; it has been suggested that it works for these conditions by promoting dopamine production in the brain, thus activating dopamine receptors that reduce the secretion of pituitary prolactin (26). Furthermore, vB6 has been shown to suppress postpartum lactation at a dosage of 600 mg/day and to reduce homocysteine serum levels at a dosage of 1,200 mg/day without any obvious ASEs after 12 weeks of use (27).

Recent methodological advancements have enabled researchers to discover previously unrecognized effects of old drugs. For example, antipsychotic agents have been shown to have surprising anticancer effects (28); and metformin has been reported to delay cognitive decline in patients with Alzheimer's disease and to alleviate chronic pain (20). Additionally, vB6 supplementation has been shown to have immune function benefits in vB6-deficient people (25), and vB6 is a potent antioxidant that quenches reactive oxygen species and thus supports cellular health (26). Although the mechanism by which vB6 reduces prolactin levels has not been delineated, vB6 therapy appears to have a favorable benefit-risk ratio given that ASEs of vB6, especially neurological ASEs, are rare (27). Based on the aforementioned findings, we were inspired to re-assess the potential efficacy of vB6 for the alleviation of AIHP.

The aim of the present study was to test the effects of high-dosage vB6, given as an adjunct treatment, on AIHP, compared to the effects of ARI, in patients with TRS. We hypothesized that high-dosage vB6 would (1) reduce prolactin levels at least as well as ARI, (2) result in a lower ASE load than ARI, and potentially (3) help to alleviate cognitive impairments in patients with TRS.

## Materials and Methods

### Participants and Study Design

A total of 200 patients with TRS were enrolled in this study from Tianjin Kangtai Hospital (a mental health treatment center) from August 1st, 2018 to July 31st, 2020. In the process of enrolling these 200 patients, we screened 260 patients, 60 of whom did not meet the inclusion criteria (below) and thus were not enrolled. The protocol for this study, which is a double-blinded randomized control trial, was approved by our hospital's institutional review board (TK-IRB-2017-C-01). All participants and their guardians signed written informed consent forms after receiving a complete explanation of the study. To avoid menstrual cycle and age effects on prolactin (29), we recruited only adult male patients between 20 and 40 years of age.

The inclusion criteria were as follows: (1) male; (2) diagnosis of TRS according to the criteria recommended by Kane [i.e., failure of at least two different ≥6-week antipsychotic medication trials at an adequate dose with objective adherence assessments; see (30)]; (3) age, 20–45 years; (4) consistent antipsychotic dosage for at least 1 month prior and during the 16-week study duration; and (5) willingness to participate and sign the informed consent form. Regarding criterion 4, it should be noted that patients were permitted to receive sedatives for the treatment of psychotic symptoms or acute agitation. The exclusion criteria were as follows: (1) allergy to vB6 or ARI; (2) diagnosis of intellectual disability, substance abuse disorder, or other psychiatric disorder besides schizophrenia; (3) history of epilepsy, head trauma, neurological disease, or systemic disease (e.g., internal organ, blood, endocrine, and metabolic diseases; mild blood glucose deviations were not considered exclusionary); (4) history of suicide attempts or serious suicidal ideation; and (5) inability to follow the study protocol.

The participants were randomized (1:1) into two groups according to a random number table. The randomization sequence was computer-generated by an independent statistician employing a randomization block size of 2. Treatment group allocations were double-blinded (to patients and investigators) and delivered in sealed envelopes in accordance with the allocation concealment method. Both evaluating researchers and those responsible for outcome analyses were blinded to the treatment allocations. One group received vB6 and the other received ARI for 16 weeks.

### Treatments

Patients were given oral vB6 at a dosage of 300 mg/12 h (600 mg/day total) (31) as an experimental therapy or oral ARI at the recommended dosage of 5 mg/12 h (10 mg/day total), which has been shown to reduce AIHP (3). Pills were taken at approximately 8:00 a.m. and 8:00 p.m. On the days that blood samples were collected; the morning pill was taken immediately after the blood draw.

### Definition of Hyperprolactinemia

In present study, hyperprolactinemia was defined as a prolactin level > 25 μg/L, in accordance with Raverot's diagnostic criteria.

### Treatment Monitoring and Efficacy Measures

All patients were monitored with the following assessments every 4 weeks. Psychotic symptoms and cognitive functioning were monitored with the Positive and Negative Syndrome Scale [PANSS (32)] and MATRICS Consensus Cognitive Battery [MCCB (33)], respectively. Blood samples were collected for liver and kidney functional enzyme, microelement (iron, calcium, copper, potassium, and sodium), and human growth hormone measurements. ASEs were monitored with the Treatment Emergent Symptom Scale [TESS (34)] and Barnes-Akathisia scale (35). Serum vB6 and ARI concentrations were determined by high-performance liquid chromatography–tandem mass spectrometry. The inter-assay precision for all analyses was <10% (36–39).

### Statistical Analysis

The data were analyzed in SAS statistical software (version 9.3, SAS Institute, Cary, NC). The data are expressed in the form of mean ± standard deviation (normally distributed data) or median ± interquartile range (non-parametric continuous-variable data) or numbers and percentages (categorical variables). Differences between the two groups were detected with Student's *t*-tests, Wilcoxon rank sum tests, and chi-square/Fisher's exact tests, as appropriate. In addition to absolute values at each timepoint, the change of serum prolactin, cognitive, psychotic symptom, and ASE scores from baseline were also compared between ARI group and vB6 group using Student's *t*-tests or Wilcoxon rank sum tests as appropriate. The efficacy analysis followed the intention-to-treat principle. To correct for missing values the last observation carried forward method was used. Mixed-effects models for repeated measures (MMRM) were employed in a sensitivity analysis aimed at evaluating the effects of time and treatment on prolactin levels after adjusting for age, education level, total drug dose, PANSS at baseline, overall MCCB at baseline triglyceride levels, and cholesterol levels. Improvement rate was defined as the proportion of participants achieving a serum prolactin level <40 ng/ml. Among the participants treated with vB6, univariate and multivariate associations of clinical-demographic characteristics with an improvement of serum prolactin level were evaluated with a logistic regression model and expressed as odds ratios (ORs) with 95% confidence interval (CIs).

## Results

### Demographic and Clinical Characteristics

**Figure 1:**
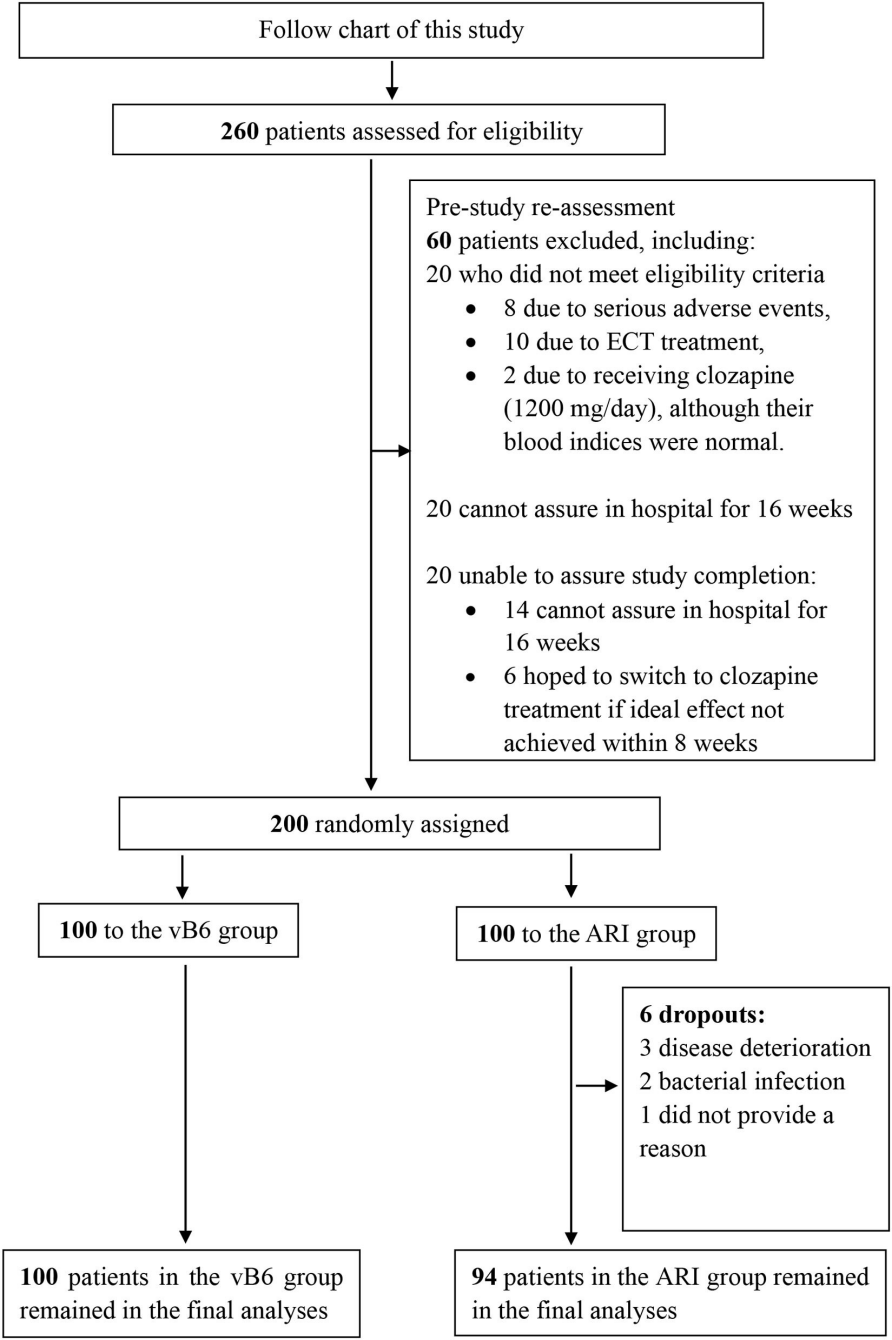
Flow chart of patient enrollment and retention in the study.

As described in the study enrollment and retention flow chart shown in Figure 1, of 260 patients with TRS who were recruited, 200 were eligible for inclusion in the study and assigned randomly to the ARI group (*N* = 100) or vB6 group (*N* = 100). A total of 6 patients in the ARI group dropped out (dropout rate, 1.5%), including 3 patients due to worsening of psychotic symptoms (they were given increased clozapine dosage and electroconvulsive therapy), two patients due to bacterial infection (they were given anti-inflammation treatment), and one patient who did not want to continue the study but did not provide a reason. No patients dropped out of the vB6 group. The full 200 patients were included in the intention-to-treat analyses, including the 6 patients who dropped out of the ARI group. The two groups were similar with respect to clinical characteristics (Table 1).

**Table 1:** Demographic and clinical characteristics at baseline, medication use, and biochemical assessment during treatment in patients with TRS by treatment group.

**Variable**		**ARI (*N =* 100)**	**vB6 (*N* = 100)**	***P***
Age, years		33.65 ± 3.03	29.99 ± 4.92	**<0.001**
Education	≤ 12 years	84 (84.0)	66 (66.0)	**0.003**
	>12 years	16 (16.0)	34 (34.0)	
Disease duration before admission, weeks		6.09 ± 1.54	6.06 ± 1.41	0.40
Psychiatric family history	No	74 (74.0)	79 (79.0)	0.89
	Yes	26 (26.0)	21 (21.0)	
Total drug dose in 16 weeks, mg		71,260.00 ± 9,424.43	70,262.00 ± 10,359.23	0.48
PANSS at baseline		91.63 ± 6.98	89.11 ± 3.56	**0.002**
Overall MCCB at baseline		29.82 ± 8.84	29.69 ± 9.17	0.92
**Baseline data test data**				
Dose, mg/d		10	600	>0.99
Blood glucose, mmol/L		5.05 ± 0.59	5.02 ± 0.43	0.71
Triglycerides, mmol/L		1.37 ± 0.29	1.57 ± 0.07	**<0.001**
Cholesterol, mmol/L		4.79 ± 0.85	5.00 ± 0.16	**0.02**
Alanine aminotransferase, U/L		47.80 ± 12.72	47.95 ± 12.66	0.93
Aspartate aminotransferase, U/L		46.60 ± 11.97	46.04 ± 11.98	0.74
Gamma-glutamyl transferase, U/L		58.20 ± 11.33	58.88 ± 11.91	0.68
Creatinine, U/L		75.06 ± 16.06	74.09 ± 16.71	0.68
Blood urea nitrogen, U/L		3.63 ± 0.53	3.60 ± 0.52	0.77
Prolactin, μg/L		89.07 ± 3.59	95.52 ± 6.30	**<0.001**

### Treatment Efficacy

**Figure 2:**
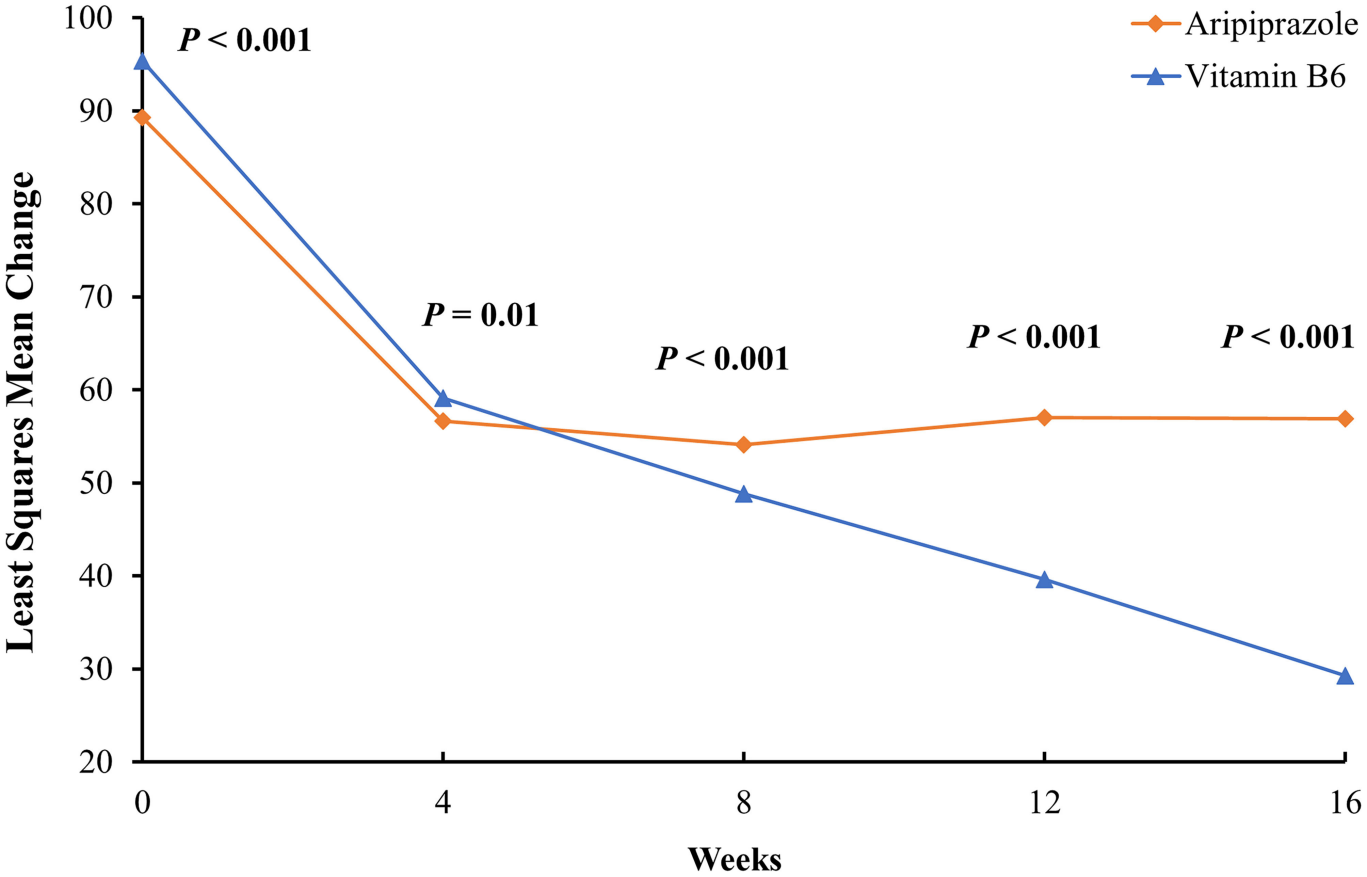
Least-squares mean change in serum prolactin levels from baseline to week 16 in men with TRS. The data were adjusted for age, education level, total drug dosage, blood triglyceride level, and blood cholesterol level. Note that although the vB6 group had, on average, higher serum prolactin levels than the ARI group, by week 4 both had achieved similarly pronounced reductions. Moreover, after week 4, prolactin levels leveled off in the ARI group while continuing to reach lower levels in the vB6 group.

From baseline levels to the end of the 16 week study period, the vB6 group showed a 68.1% reduction in prolactin levels from 95.52 ± 6.30 μg/L to 30.43 ± 18.65 μg/L while the ARI group showed a 37.4% reduction from 89.07 ± 3.59 μg/L to 55.78 ± 7.39 μg/L. From baseline to week 4, both groups demonstrate similarly steep reductions in prolactin levels. However, the trends diverged subsequently, especially after week 8 when the efficacy of ARI plateaued. Meanwhile, vB6 continued to further reduce prolactin levels through week 16. MMRM showed a significant group × time interaction effect on prolactin levels (*F* = 112.63, *p* < 0.001). The statistical results of inter-group comparisons are reported in Table 2 and Figure 2.

**Table 2:** Prolactin levels from baseline through week 16 in men with TRS.

**Variable**		**ARI (*N* = 100)**	**vB6 (*N* = 100)**	***P***
Change in serum prolactin from baseline (μg/L)	Week 4	−33.01 ± 8.05	−35.80 ± 13.71	0.08
	Week 8	−35.70 ± 8.01	−45.97 ± 14.47	**<0.001**
	Week 12	−33.62 ± 8.08	−54.33 ± 16.41	**<0.001**
	Week 16	−33.29 ± 7.80	−65.09 ± 19.48	**<0.001**
Proportion with prolactin <40 μg/ml	Week 4	1 (1.0)	7 (7.0)	0.06
	Week 8	2 (2.0)	22 (22.0)	**<0.001**
	Week 12	1 (1.0)	48 (48.0)	**<0.001**
	Week 16	2 (2.0)	67 (67.0)	**<0.001**

*MMRM showed a significant group × time interaction effect on prolactin level (F = 112.63, p < 0.001)*.

### Factors Associated With Prolactin Reduction

Multivariate logistic regression model analysis revealed four factors as being significantly related to prolactin level reduction to <40 μg/L at week 16: education level; total drug dose at the conclusion of week 16; baseline triglyceride level; and baseline prolactin level. The resultant ORs, CIs, and *p-*values are reported in Table 3 and Supplementary Table 1.

**Table 3:** Factors associated with improvement of serum prolactin level to <40 μg/ml at week 16 among participants treated with vB6 as indicated by multivariate logistic regression modeling.

**Factor**	**OR (95% CI)**	***P***
Education level (>12 years vs. ≤ 12)	2.79 (1.34–5.80)	**0.006**
Total drug dose in 16 weeks	0.10 (0.01–0.85)	**0.04**
Triglycerides at baseline	23.47 (3.02–182.43)	**0.003**
Prolactin at baseline	1.11 (1.04–1.17)	**<0.001**

### Psychometric Measures

Psychometric scores obtained for the two groups are shown in Table 4. The vB6 group had significantly better (lower) PANSS scores than the ARI group at baseline, week 4, and week 16. The vB6 group exhibited a 17.8% reduction in PANSS scores from baseline to week 16, while the ARI group exhibited only an 11.96% reduction (*p* < 0.001). MCCB total scores were similar between the two groups at baseline, but higher in the vB6 group than in the ARI group by week 16.

**Table 4:** Cognitive, psychotic symptom and ASE scores at baseline and after 16 weeks in patients with TRS.

**Psychometric measure**		**ARI (*N* = 100)**	**vB6 (*N* = 100)**	***P***
**Cognitive (sub)scale**				
Brief Assessment of Cognition in Schizophrenia-Symbol Coding	Baseline	31.81 ± 9.68	31.74 ± 9.25	0.96
	Week 16	31.28 ± 9.04	30.23 ± 9.29	0.42
	Change	−0.53 ± 11.62	−1.51 ± 2.00	0.41
Fluency	Baseline	30.26 ± 7.54	29.16 ± 9.47	0.36
	Week 16	30.26 ± 7.34	30.39 ± 8.87	0.91
	Change	0.00 ± 3.56	1.23 ± 5.43	0.06
Trail-making test, part A	Baseline	29.68 ± 7.06	29.58 ± 9.02	0.93
	Week 16	29.33 ± 8.32	31.44 ± 8.19	0.07
	Change	−0.35 ± 5.82	1.86 ± 5.00	0.004
Attention/vigilance	Baseline	29.19 ± 8.22	28.09 ± 9.03	0.37
	Week 16	30.40 ± 8.23	30.61 ± 8.54	0.86
	Change	1.21 ± 4.89	2.52 ± 5.87	0.09
Wechsler Memory Scale-III, Symbol Span	Baseline	29.91 ± 8.23	28.58 ± 8.60	0.27
	Week 16	30.11 ± 8.16	31.46 ± 8.27	0.25
	Change	0.20 ± 5.23	2.88 ± 6.74	0.002
Letter number sequencing	Baseline	31.03 ± 8.45	30.99 ± 8.61	0.97
	Week 16	29.11 ± 8.58	29.98 ± 8.52	0.47
	Change	−1.92 ± 2.55	−1.01 ± 1.79	0.004
Hopkins Verbal Learning Test–Revised	Baseline	27.86 ± 8.44	30.65 ± 9.39	0.03
	Week 16	29.91 ± 8.39	29.66 ± 8.73	0.84
	Change	2.05 ± 3.94	−0.99 ± 1.77	<0.001
Brief Visuospatial Memory Test–Revised	Baseline	28.73 ± 8.43	30.58 ± 9.10	0.14
	Week 16	30.59 ± 8.55	30.58 ± 9.10	0.99
	Change	1.86 ± 3.98	0.00 ± 0.00	<0.001
Neuropsychological assessment battery mazes	Baseline	29.86 ± 8.39	29.88 ± 8.91	0.99
	Week 16	30.39 ± 9.31	28.88 ± 8.90	0.24
	Change	0.54 ± 3.30	−1.00 ± 1.20	<0.001
Mayer-Salovey-Caruso Emotional Intelligence Test, Managing Emotions	Baseline	30.34 ± 9.22	30.53 ± 9.58	0.89
	Week 16	27.97 ± 9.94	29.56 ± 9.46	0.25
	Change	−2.37 ± 4.35	−0.97 ± 1.35	<0.001
MCCB total score	Baseline	29.82 ± 8.84	29.69 ± 9.17	0.92
	Week 16	28.20 ± 8.90	32.64 ± 9.65	<0.001
	Change	−1.62 ± 2.01	2.95 ± 7.29	<0.001
**Psychotic symptoms**				
PANSS	Baseline	91.63 ± 6.98	89.11 ± 3.56	0.002
	Week 4	85.06 ± 10.57	88.66 ± 3.71	0.002
	Week 8	82.4 ± 12.49	82.30 ± 8.64	0.95
	Week 12	80.2 ± 14.53	78.27 ± 11.03	0.29
	Week 16	80.67 ± 14.59	73.27 ± 17.19	0.001
Change of PANSS from baseline	Week 4	−6.57 ± 10.31	−0.45 ± 2.78	<0.001
	Week 8	−9.23 ± 10.95	−6.81 ± 9.53	0.10
	Week 12	−11.43 ± 12.34	−10.84 ± 11.72	0.73
	Week 16	−10.96 ± 14.11	−15.84 ± 17.88	0.03
**ASEs**				
Barnes-Akathisia	Baseline	2.83 ± 4.15	1.93 ± 3.96	0.12
	Week 4	4.02 ± 5.10	2.51 ± 4.40	0.03
	Week 8	4.42 ± 5.71	2.44 ± 4.77	0.008
	Week 12	4.66 ± 5.96	2.47 ± 4.76	0.005
	Week 16	4.45 ± 5.84	2.32 ± 4.65	0.005
Change of Barnes-Akathisia from baseline	Week 4	1.19 ± 2.44	0.58 ± 1.74	0.04
	Week 8	1.59 ± 2.81	0.51 ± 1.98	0.002
	Week 12	1.83 ± 3.08	0.54 ± 1.95	<0.001
	Week 16	1.62 ± 3.37	0.39 ± 2.12	0.002
TESS	Baseline	1.88 ± 0.54	1.77 ± 0.47	0.12
	Week 4	1.89 ± 0.55	1.75 ± 0.50	0.06
	Week 8	1.89 ± 0.55	1.64 ± 0.54	0.001
	Week 12	1.88 ± 0.54	1.60 ± 0.53	<0.001
	Week 16	1.89 ± 0.55	1.63 ± 0.56	0.001
Change of TESS from baseline	Week 4	0.01 ± 0.10	−0.02 ± 0.20	0.18
	Week 8	0.01 ± 0.10	−0.13 ± 0.37	<0.001
	Week 12	0.00 ± 0.00	−0.17 ± 0.38	<0.001
	Week 16	0.01 ± 0.36	−0.14 ± 0.43	0.008
AEs	No	89 (89.0)	94 (94.0)	0.20
	Yes	11 (11.0)	6 (6.0)	
	Urinary incontinence	4 (4.0)	0 (0.0)	
	Epilepsy	2 (2.0)	0 (0.0)	
	Numbness of limbs	0 (0.0)	2 (2.0)	
	Instability of gait	0 (0.0)	2 (2.0)	
	Headache	2 (2.0)	0 (0.0)	
	Other	2 (2.0)	2 (2.0)	

With respect to cognitive performance, the vB6 group showed an average increase in MCCB total score of 9.94% during the study period, while the ARI group showed a decrease of 5.43% (*p* < 0.001). The only MCCB sub-score that differed significantly between the groups was the working memory score. None of the patients in the vB6 group experienced a clinically significant deterioration in psychotic symptoms (PANSS score increase of ≥15%). Meanwhile, 4 patients in the vB6 group experienced a clinically significant improvement in psychotic symptoms (≥50% reduction in PANSS score). In the patients in the ARI group, 4 patients experienced a clinically significant deterioration in psychotic symptoms (including 3 who dropped out) and 2 patients showed a clinically significant improvement.

### Safety and Tolerance

According to Barnes-Akathisia scale and TESS scores (Table 4), the vB6 group experienced lesser ASEs than the ARI group. The most frequently observed ASEs overall were drowsiness (73.5%, 147/200), akathisia (64.5%, 129/200), dizziness (56.5%, 113/200), constipation (58.50%, 117/200), hand tremor (50.5%, 101/200), salivation (47.0%, 94/200), transient arrhythmia (31.5%, 53/200), and orthostatic hypotension (23.5%, 47/200). The incidences of these ASEs did not differ significantly between the two groups (data not shown). Thus, the aforementioned significant differences in scale scores can thus be attributed in large part to the greater severity of ASEs in the ARI group, consistent with our general clinical observations. More importantly, blood sugar, liver function, and kidney function did not differ significantly between two groups (Supplementary Table 2). However, levels of triglycerides and cholesterol did differ significantly between the groups, with lower levels of both being found in the vB6 group (Supplementary Table 2).

## Discussion

In the present study, our results demonstrate, for the first time to our knowledge, that administration of high-dosage vB6 as an adjunct treatment reduces elevated prolactin in patients with TRS as effectively as ARI in the first 4 weeks of treatment and more effectively than ARI thereafter. Our data also suggest that vB6 had a beneficial effect on cognition. Our data also suggest that vB6 had a beneficial effect on cognition. No serious ASEs were observed in this study in either group. Overall, we observed an encouraging safety and efficacy profile for vB6 in TRS patients needing AIHP treatment.

The mechanism underlying improved PANSS scores in the vB6 group are unknown. It is possible that these benefits are related to the neuroprotective and anti-inflammatory effects of vB6. Concurrent improvements in PANSS scores in the ARI group could reflect an augmentation effect of ARI relative to the previous antipsychotic agent that the patient had been using. Similar ASEs emerged in the two groups, including drowsiness, constipation, dry mouth, akathisia, hydro-stomia, transient arrhythmia, and orthostatic hypotension. Thus, treating physicians should be vigilant in looking for signs of ASEs. Notably, it is important to treat constipation, which can lead to hyperammonemia (40, 41).

According to the estrogen protection hypothesis of AIHP, increases in estrogen levels, which can modulate psychotic symptoms in schizophrenia (40, 42–45), lead to competitive antagonism of inhibitory type 2 dopamine (D2) receptors in the pituitary. Normally, dopamine binding to D2 receptors inhibits prolactin secretion from prolactin secreting cells. Thus, antagonism of D2 receptors results in increased secretion of prolactin (7, 46–51). The stress-prolactin-dopamine hypothesis is informed by observations indicating that prolactin itself can act as a stress hormone, being released in response to stressful stimulation. This stress-induced prolactin release may then trigger a positive feedback mechanism in which increased dopamine release triggers further prolactin elevation, while also potentially worsening psychotic symptoms (7, 47, 50, 52). Elevations in prolactin may then suppress gonadal estrogen production as a negative feedback adaption. Interestingly, schizophrenic patients with hyperprolactinemia have been reported to have increased pituitary volumes, consistent with the stress-prolactin-dopamine hypothesis (52–56). These two hypotheses converge in suggesting that D2 receptor activation in prolactin cells in the pituitary plays a pivotal role in AIHP.

Supplemental vB6 can enhance hypothalamic/pituitary dopaminergic neuron activity, increase dopamine levels, and thus reduce competitive antipsychotic drug occupation of D2 receptors while enabling dopamine to inhibit secretion from prolactin cells (57, 58). In another study, vB6 was reported to inhibit prolactin secretion partially via a dopamine-independent mechanism (59–61). ARI is a partial D2 receptor agonist with effects that are often sufficient to maintain prolactin within physiological levels (62). Hence, based on the available evidence, it appears that vB6 influences prolactin levels via a mechanism or mechanisms different from that of ARI, such as enabling dopamine to inhibit prolactin hypersecretion and perhaps additionally via a dopamine-independent mechanism.

### Limitations

There are a number of notable limitations in the present study. First, the average baseline prolactin level was higher and the average age was younger in the vB6 group than in the ARI group. Because patients were assigned to groups randomly and this was a double-blinded study, we could not match subjects for prolactin levels or age across the two groups. Thus, future research with a different study design should address these factors. Second, we only monitored whether microelements were within a normal range or not. More detailed blood concentration analyses were beyond the scope of our protocol. However, because blood microelement concentrations may influence efficacy or ASE risk, more detailed micronutrient analyses should be performed in this patient population. Third, because our focus was on AIHP in patients with TRS, rather than in schizophrenic patients whose psychotic symptoms are well-controlled, some patients dropped out due to symptom worsening. Fourth, because the patients had TRS, they were taking relatively high doses of antipsychotics compared to the average schizophrenic patient and thus had quite high prolactin levels, relative to other studies in the literature. The efficacy of vB6 in treatment-responsive schizophrenic patients remains to be determined. Fifth, although we did not find that microelement or growth hormone levels were significantly altered in the vB6 group, we only monitored patients for 16 weeks. Longer-term monitoring data are thus needed. Sixth, we did not divide the patients into subgroups according to the particular antipsychotic agent they were using. Seventh, we did not control for stress or stress hormone levels in our analysis. Eighth, because this was a single-center study, it is not known how well our findings would generalize to other clinical contexts. Finally, although we adopted a previously used TRS criterion, TRS itself is a descriptive category that does not reflect underlying genetic or environmental causes of treatment resistance.

## Conclusion

Compared to ARI, vB6 was more effective for reducing prolactin levels and was associated with lesser ASEs in TRS patients with AIHP over 16 weeks. Additionally, vB6 may have cognitive benefits in this patient population. We conclude that vB6 is a promising candidate adjunct AIHP treatment, though longer-term monitoring of effects on microelement and growth hormone levels are needed.

## Data Availability Statement

The raw data supporting the conclusions of this article will be made available by the authors, without undue reservation.

## Ethics Statement

The studies involving human participants were reviewed and approved by Tianjin Kangtai Hospital‘s institutional review board. The patients/participants provided their written informed consent to participate in this study.

## Author Contributions

CZhu had full access to all of the data in the study and takes responsibility for the integrity of the data and the accuracy of the data analysis. CZhu, CL, and YX: concept and design. CZho, HT, JC, JL, TF, CY, WY, QL, AY, SX, BL, and YC: acquisition, analysis, or interpretation of data. HT, CZhu, and HW: drafting of the manuscript. CZhu, HW, YX, and CZ: critical revision of the manuscript for important intellectual content. HW and CZhu: statistical analysis. CZhu: obtained funding. AY, HT, BL, and YC: administrative, technical, or material support. HW and CZhu: supervision. All authors contributed to the article and approved the submitted version.

## Conflict of Interest

The authors declare that the research was conducted in the absence of any commercial or financial relationships that could be construed as a potential conflict of interest.

## Publisher's Note

All claims expressed in this article are solely those of the authors and do not necessarily represent those of their affiliated organizations, or those of the publisher, the editors and the reviewers. Any product that may be evaluated in this article, or claim that may be made by its manufacturer, is not guaranteed or endorsed by the publisher.

## Abbreviations

vB6: vitamin B6
AIHP: antipsychotic-induced hyperprolactinemia
TRS: treatment-resistant schizophrenia
ARI: aripiprazole
PANSS: Positive and Negative Syndrome Scale
MCCB: MATRICS Consensus Cognitive Battery
ASEs: adverse secondary effects
TESS: Treatment Emergent Symptom Scale
ORs: odds ratios
CIs: confidence interval.
