# Supplementary material for: Safety and Efficacy of High-Dose Vitamin B6 as an Adjunctive Treatment for Antipsychotic-Induced Hyperprolactinemia in Male Patients With Treatment-Resistant Schizophrenia
Source: Front Psychiatry. 2021 Aug 26;12:681418. doi: 10.3389/fpsyt.2021.681418 (PMC8426548; doi:10.3389/fpsyt.2021.681418)
Supplement: Supplementary file 1 [file Table_1.DOC]

**Supplemental Table 1.** Univariate logistic regression analysis of factors associated with improvement of serum prolactin level (to <40 ng/ml at week 16) among TRS patients treated with vB6.

| **Factor** | | **≥40 ng/ml** | **<40 ng/ml** | **OR (95% CI)** | ***P*** |
| --- | --- | --- | --- | --- | --- |
| Age | | 32.46 ± 4.34 | 30.61 ± 4.49 | 0.91 (0.85–0.97) | **.006** |
| Education | ≤12 years | 107 (71.3) | 43 (28.7) | 1.0 | **.003** |
|  | >12 years | 24 (48.0) | 26 (52.0) | 2.70 (1.40–5.21) |  |
| Disease duration before admission, weeks | | 6.05 ± 1.51 | 6.12 ± 1.41 | 1.04 (0.85–1.26) | .74 |
| Psychiatric family history | No | 94 (61.4) | 59 (38.6) | 1.0 | **.03** |
|  | Yes | 37 (78.7) | 10 (21.3) | 0.43 (0.20–0.93) |  |
| Total 16-week drug dose | | 71,464.12 ± 9,516.7 | 69,426.09 ± 10,505.96 | 0.26 (0.04–1.73) ^¶^ | .16 |
| Baseline PANSS score | | 90.85 ± 6.34 | 89.45 ± 4.00 | 0.96 (0.91–1.01) | .10 |
| Baseline MCCB total score | | 29.50 ± 8.70 | 30.23 ± 9.55 | 1.01 (0.98–1.04) | .58 |
| *Baseline blood test measure* | |  |  |  |  |
| Dose, mg/day | | 481.17 ± 322.70 | 452.52 ± 40.03 | 0.47 (0.05–4.47) ^¶^ | .51 |
| Blood glucose, mmol/L | | 5.02 ± 0.55 | 5.06 ± 0.43 | 1.13 (0.63–2.03) | .67 |
| Triglycerides, mmol/L | | 1.42 ± 0.27 | 1.56 ± 0.09 | 41.18 (5.86–289.17) | **<.001** |
| Cholesterol, mmol/L | | 4.85 ± 0.75 | 4.98 ± 0.17 | 1.44 (0.86–2.4) | .17 |
| Alanine aminotransferase, U/L | | 48.14 ± 12.57 | 47.38 ± 12.91 | 1.00 (0.97–1.02) | .69 |
| Aspartate aminotransferase, U/L | | 46.16 ± 11.83 | 46.62 ± 12.25 | 1.00 (0.98–1.03) | .79 |
| Gamma-glutamyl transferase, U/L | | 58.12 ± 12.00 | 59.33 ± 10.84 | 1.01 (0.98–1.04) | .48 |
| Creatinine, U/L | | 74.48 ± 16.23 | 74.75 ± 16.70 | 1.00 (0.98–1.02) | .91 |
| Blood urea nitrogen, U/L | | 3.62 ± 0.54 | 3.61 ± 0.50 | 0.95 (0.55–1.66) | .86 |
| Prolactin, μg/L | | 90.89 ± 5.85 | 94.96 ± 5.54 | 1.13 (1.07–1.19) | **<.001** |

^¶^ Calculated after the logarithm transformation.

**Supplemental Table 2.** Biochemical assessments in male patients with TRS treated with vB6.

| **Blood sample measurement** | | **ARI (N = 100)** | **vB6 (N = 100)** | ***P*** |
| --- | --- | --- | --- | --- |
| Drug, mg/day | Baseline | 490.55 ± 368.63 | 452.02 ± 39.76 | .30 |
|  | Week 4 | 454.39 ± 40.32 | 454.60 ± 39.78 | .97 |
|  | Week 8 | 457.26 ± 38.05 | 457.16 ± 40.01 | .99 |
|  | Week 12 | 456.52 ± 39.12 | 455.99 ± 39.62 | .92 |
|  | Week 16 | 455.43 ± 39.40 | 455.06 ± 39.01 | .95 |
| Blood glucose, mmol/L | Baseline | 5.05 ± 0.59 | 5.02 ± 0.43 | .71 |
|  | Week 4 | 5.25 ± 0.73 | 5.10 ± 0.35 | .06 |
|  | Week 8 | 5.34 ± 0.86 | 5.24 ± 0.54 | .35 |
|  | Week 12 | 5.68 ± 0.69 | 5.18 ± 0.35 | **<.001** |
|  | Week 16 | 5.18 ± 0.63 | 5.13 ± 0.32 | .52 |
| Triglycerides, mmol/L | Baseline | 1.37 ± 0.29 | 1.57 ± 0.07 | **<.001** |
|  | Week 4 | 1.37 ± 0.31 | 1.59 ± 0.09 | **<.001** |
|  | Week 8 | 1.29 ± 0.35 | 1.58 ± 0.13 | **<.001** |
|  | Week 12 | 1.84 ± 0.46 | 1.58 ± 0.09 | **<.001** |
|  | Week 16 | 1.25 ± 0.35 | 1.59 ± 0.12 | **<.001** |
| Cholesterol, mmol/L | Baseline | 4.79 ± 0.85 | 5.00 ± 0.16 | **.02** |
|  | Week 4 | 4.99 ± 0.95 | 5.23 ± 0.28 | **.02** |
|  | Week 8 | 5.59 ± 0.72 | 5.28 ± 0.39 | **<.001** |
|  | Week 12 | 5.67 ± 0.67 | 5.29 ± 0.40 | **<.001** |
|  | Week 16 | 5.40 ± 0.86 | 5.22 ± 0.28 | .06 |
| Alanine aminotransferase, U/L | Baseline | 47.80 ± 12.72 | 47.95 ± 12.66 | .93 |
|  | Week 4 | 62.99 ± 7.63 | 63.24 ± 7.59 | .82 |
|  | Week 8 | 69.05 ± 11.4 | 68.86 ± 11.39 | .91 |
|  | Week 12 | 64.60 ± 9.19 | 65.01 ± 9.31 | .75 |
|  | Week 16 | 84.23 ± 10.91 | 83.57 ± 11.09 | .67 |
| Aspartate aminotransferase, U/L | Baseline | 46.60 ± 11.97 | 46.04 ± 11.98 | .74 |
|  | Week 4 | 64.20 ± 7.41 | 65.06 ± 7.69 | .42 |
|  | Week 8 | 69.19 ± 2.57 | 69.04 ± 12.66 | .93 |
|  | Week 12 | 82.87 ± 11.63 | 82.44 ± 11.70 | .79 |
|  | Week 16 | 79.49 ± 12.64 | 78.36 ± 13.37 | .54 |
| Gamma-glutamyl transferase, U/L | Baseline | 58.20 ± 11.33 | 58.88 ± 11.91 | .68 |
|  | Week 4 | 71.49 ± 12.21 | 71.50 ± 12.21 | .99 |
|  | Week 8 | 79.66 ± 14.14 | 79.95 ± 13.75 | .88 |
|  | Week 12 | 80.53 ± 14.39 | 79.74 ± 14.23 | .70 |
|  | Week 16 | 72.58 ± 16.12 | 72.73 ± 15.11 | .95 |
| Creatinine, U/L | Baseline | 75.06 ± 16.06 | 74.09 ± 16.71 | .68 |
|  | Week 4 | 64.15 ± 16.84 | 63.91 ± 16.78 | .92 |
|  | Week 8 | 59.06 ± 13.11 | 58.39 ± 13.36 | .72 |
|  | Week 12 | 56.80 ± 14.2 | 56.86 ± 14.01 | .98 |
|  | Week 16 | 59.02 ± 2.01 | 57.98 ± 12.57 | .55 |
| Blood urea nitrogen, U/L | Baseline | 3.63 ± 0.53 | 3.60 ± 0.52 | .77 |
|  | Week 4 | 3.29 ± 0.61 | 3.28 ± 0.58 | .87 |
|  | Week 8 | 2.97 ± 0.58 | 2.99 ± 0.55 | .85 |
|  | Week 12 | 2.96 ± 0.62 | 2.93 ± 0.60 | .72 |
|  | Week 16 | 3.06 ± 0.59 | 3.03 ± 0.56 | .72 |
